# Contributions to the discussion on societal impact assessment in graduate programs in dentistry in Brazil

**DOI:** 10.1590/0103-644020256764

**Published:** 2025-10-24

**Authors:** Mauro Henrique Nogueira Guimarães de Abreu, André Luiz Brasil Varandas Pinto, Cecilia Claudia Costa Ribeiro de Almeida, Vicente Castelo Branco Leitune, José Mauro Granjeiro, Altair Antoninha Del Bel Cury, Antonio Gomes Souza, Manoel Damião

**Affiliations:** 1 Departamento de Odontologia Social e Preventiva, Faculdade de Odontologia, Universidade Federal de Minas Gerais Belo Horizonte, MG, Brasil; 2 Centre for Science and Technology Studies, Universidade de Leiden, Leiden, Holanda , Coordenação de Aperfeiçoamento de Pessoal de Nível Superior (CAPES), Brasília, Brasil; 3 Departamento de Odontologia, Universidade Federal do Maranhão, São Luiz, MA, Brasil; 4 Faculdade de Odontologia, Universidade Federal do Rio Grande do Sul, Porto Alegre, RS, Brasil; 5 Instituto Nacional de Metrologia, Qualidade e Tecnologia (Inmetro). Faculdade de Odontologia, Universidade Federal Fluminense, Niterói, RJ, Brasil; 6 Faculdade de Odontologia, Universidade de Campinas, Piracicaba, SP, Brasil; 7 Departmento de Física, Universidade Federal do Ceará, Fortaleza, CE, Brazil, Coordenação de Aperfeiçoamento de Pessoal de Nível Superior (CAPES), Brasília, Brasil; 8 Faculdade de Odontologia, Universidade de São Paulo, Ribeirão Preto, SP, Brasil

**Keywords:** Education, Biological and Biomedical Sciences, Graduate, Education, dental, Bibliometrics

## Abstract

This article aims to discuss the scientific and societal impact of Brazilian graduate programs in dentistry and propose an impact assessment framework considering recent changes in the national evaluation model. The study highlights the high scientific productivity and global relevance of Brazilian dental research by examining bibliometric data from the Scopus and Scival platforms. The bibliometric map of abstracts from theses and dissertations developed at graduate programs in dentistry between 2013 and 2022 leads to at least three thematic clusters, suggesting consistency and diversity in the knowledge produced. Three consolidated frameworks were reviewed to explore the societal impact: the Payback Model, SIAMPI, and CIROP. Based on these theoretical frameworks, the article proposes a set of illustrative indicators based on the Kellogg Foundation’s logic model and the Strategy Evaluation Protocol (SEP), aligned with the Sustainable Development Goals (SDGs). These indicators may support the self-assessment of graduate programs and feed into the national evaluation systems. Integrating these elements enables a multidimensional view of impact, capturing excellence in knowledge production and contributions to public health, innovation, and equity promotion. The proposed framework is flexible and adaptable to different institutional missions and regional realities, and is particularly relevant in a country where academic and professional graduate programs coexist. The article concludes by emphasizing the importance of including scientific and societal impact in evaluation processes, thus strengthening the connection between research, health policies, and Society's needs.

## Resumo

Este artigo tem como objetivo analisar o impacto científico e social dos programas brasileiros de pós-graduação em odontologia, considerando as recentes mudanças nos modelos nacionais de avaliação. Com base em dados bibliométricos das plataformas Scopus e SciVal, bem como no mapeamento temático usando o VOSviewer, o estudo destaca a alta produtividade científica e a relevância global da pesquisa odontológica brasileira. Para explorar o impacto social, três estruturas consolidadas foram revisadas: o Modelo Payback, o SIAMPI e o CIROP. Esses modelos enfatizam a necessidade de abordagens diversas e sensíveis ao contexto para avaliar a pesquisa além dos resultados acadêmicos. Com base nesses fundamentos teóricos, o artigo propõe um conjunto de indicadores ilustrativos alinhados com os Objetivos de Desenvolvimento Sustentável (ODS), o modelo lógico da Fundação Kellogg e o Protocolo de Avaliação de Estratégias (SEP). Esses indicadores visam apoiar a autoavaliação dos programas de pós-graduação e informar os sistemas nacionais de avaliação. Um mapa bibliométrico de resumos de teses e dissertações produzidas por programas de pós-graduação em odontologia entre 2013 e 2022 foi gerado usando o software VOSviewer. A análise identificou pelo menos três grupos temáticos, sugerindo consistência e diversidade no conhecimento produzido. A integração desses elementos permite uma visão multidimensional do impacto, capturando tanto a excelência na produção de conhecimento quanto as contribuições para a saúde pública, a inovação e a equidade. A estrutura proposta é flexível e adaptável a diferentes missões institucionais, o que é particularmente relevante em um país onde coexistem programas de pós-graduação acadêmicos e profissionais. Seria benéfico considerar a inclusão de estudos de caso sobre produtos com impacto social identificados pelos programas de pós-graduação. O artigo conclui enfatizando a importância de incluir o impacto científico e social nos processos de avaliação, fortalecendo a conexão entre pesquisa, políticas de saúde e necessidades da comunidade.

**Figure:**
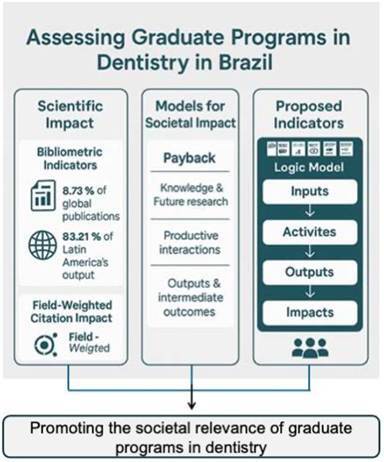

## Introduction

According to data from the Brazilian Agency for Support and Evaluation of Graduate Education (CAPES), the number of graduate programs^1^ (PPG) in Brazil has grown by 48.45% in the past decade^2^. In 2020, the country had 4,650 PPGs, 99 of which were in dentistry. While Brazil has emerged as a major contributor to scientific output-ranking at 12th position globally in overall scientific publications according to the Scopus database-its performance in dentistry is particularly noteworthy: between 2009 and 2020, it placed second worldwide in the number of published articles, with Brazilian researchers addressing a variety of pivotal topics in dental science. In Brazil, almost all the scientific research is performed within the graduate programs.

With this growth, the next challenge is to update the national evaluation system of graduate education for the quadrennial cycle that began in 2025, adopting a more qualitative model and assessing the regional reach of research and its societal impact. Moving away from the previous growth-oriented, quantitative model, this new framework emphasizes the quality of PPG activities by focusing on the programs’ pedagogical project alongside three key dimensions: 1) The Program, 2) Training and intellectual production, and 3) Impact (local, regional, national, and international). This shift also requires a deeper understanding of scientific impact, including the use of bibliometric indicators to assess the relevance and reach of academic production.

Brazilian PPGs produce the country's scientific output through article publishing ^1^. Bibliometric indicators, such as publication output, citation counts, and field-weighted citation impact (FWCI), help to capture the relevance and dissemination of scientific knowledge, thus supporting evidence-based policy, innovation, and clinical practice. Science's impact can be measured in multiple ways, with scientific and technological impacts serving as the foundation for broader societal benefits. Therefore, it is essential to discuss how the scientific knowledge emerging from master's and doctoral courses impacts Society.

Research can generate economic, social, political, organisational, educational, public health, environmental, and symbolic impacts ^2^. Lesjak ^3^ further asserts that the ultimate goal of science is to foster socio-economic impact, encompassing national security, economic and social development, public well-being, and environmental protection, while recognising the challenge in evaluating such a complex and multifaceted subject. The evaluation should be designed to balance the assessment of scientific advancements through standard science metrics and the assessment of societal impact.

In dentistry research, the main objective is to improve oral health at both the individual and population levels. The FDI World Dental Federation-one of the oldest dental associations worldwide-recently defined oral health as “a multifaceted concept involving the ability to speak, smile, smell, taste, touch, chew, swallow, and express a full range of emotions through facial expressions with confidence and without pain, discomfort, or disease of the craniofacial complex” ^4^. Assessing the scientific impact of graduate programs in dentistry can help gauge the quality of their output and their influence on other health fields. Similarly, examining the societal impact of these programs may reveal how the knowledge they generate has improved oral health and reduced inequalities in the different social groups.

This article aims to discuss and critically analyse the scientific and societal impact of Brazilian graduate programs in dentistry by combining bibliometric indicators, thematic mapping of research outputs, and an impact assessment framework based on international models. The goal is to contribute to developing more comprehensive evaluation systems that reflect the missions and programs’ diverse missions and social relevance of the Brazilian graduate programs in dentistry.

### Research quality and scientific impact

Brazilian dental science occupies a prominent position in the global research landscape. According to data obtained through the SciVal platform, between 2009 and 2020, the country ranked second worldwide in the number of published dental articles. From 1996 to 2023, Brazilian authors accounted for 8.73% of all global publications in dentistry and 83.21% of Latin America's output, summing up about 35,000 scientific papers. These results demonstrate the significant contribution of Brazilian graduate programs to advancing oral health research worldwide.

Evaluating the scientific impact of graduate programs is a great challenge. Scientific outputs-such as journal articles, patents, technological developments, books, standards, and more-can impact clinical dental practice and public health. The impact can occur in the short or long term, as basic research often requires time to yield tangible outcomes. In response, several metrics have been devised to measure the impact and relevance of these scientific outputs.

The scientific impact of newly generated knowledge is closely linked to the publication of research findings. The number of published papers is frequently used as an indicator of researchers’ productivity and prestige. Brazil has emerged as a key contributor to global scientific output, ranking 12th and 14th worldwide in scientific publications. From 2009 to 2020, Brazilian researchers have contributed to different topics, which led Brazil to be ranked as the second country in the world with the most published papers in the dentistry field. However, this focus on publication counts only can encourage ‘salami slicing’-breaking studies into smaller, lower-quality articles. Moreover, publication volumes vary substantially across different fields. Metrics incorporating citation data may offer a more accurate means of evaluating researchers and institutions.

The impact factor of a journal is a widely utilised metric that reflects the average number of citations per article. The highest CiteScore and Journal Impact Factor in dentistry are 34.1 and 17.5, respectively, placing dental journals among the uppermost percentile in academic publishing. Nonetheless, these metrics measure the journal’s overall influence rather than the relevance of individual papers. For individual evaluations, the number of citations per paper can be more revealing, as it highlights the specific influence of a paper within its field. Citation patterns also differ markedly between subject areas, with some fields naturally accruing more citations than others. This variance complicates direct comparisons across diverse subject areas of dentistry.

The h-index and h5-index gauge both productivity and citation impact by identifying the most significant number of publications that have received at least the same number of citations. However, like simple citation counts, these measures depend on field-specific characteristics, which can undermine comparisons between researchers, PPG, or institutions. A more field-sensitive metric is the FWCI, which calculates the ratio of actual citations received to the average expected citations in the same field. Despite its value, FWCI tends to favour more senior researchers, as it generally increases over time. With 45,955 citations (an average of 5.1 per publication), Brazilian dental science has an FWCI of 1.08, considering the 2021 to 2024 period. Complementary indicators such as the Mean Normalized Citation Score (MNCS) and the percentage of publications in the top 10% most cited globally (%PPTOP10) could provide additional insights into the quality and influence of Brazilian dental research. These metrics help minimize biases related to field and publication year, thus offering a more balanced comparison across PPG and institutions.

Given the range of available metrics for measuring the scientific impact of researchers and institutions, it is essential to consider their respective advantages and limitations. Discrepancies across disciplines, types of outputs, and other influential factors should be taken into account when assessing scientific contributions. When used alongside other quantitative and qualitative measures, FWCI stands out as a particularly informative metric, thus providing a more comprehensive perspective on scientific impact.

**Figure 1:**
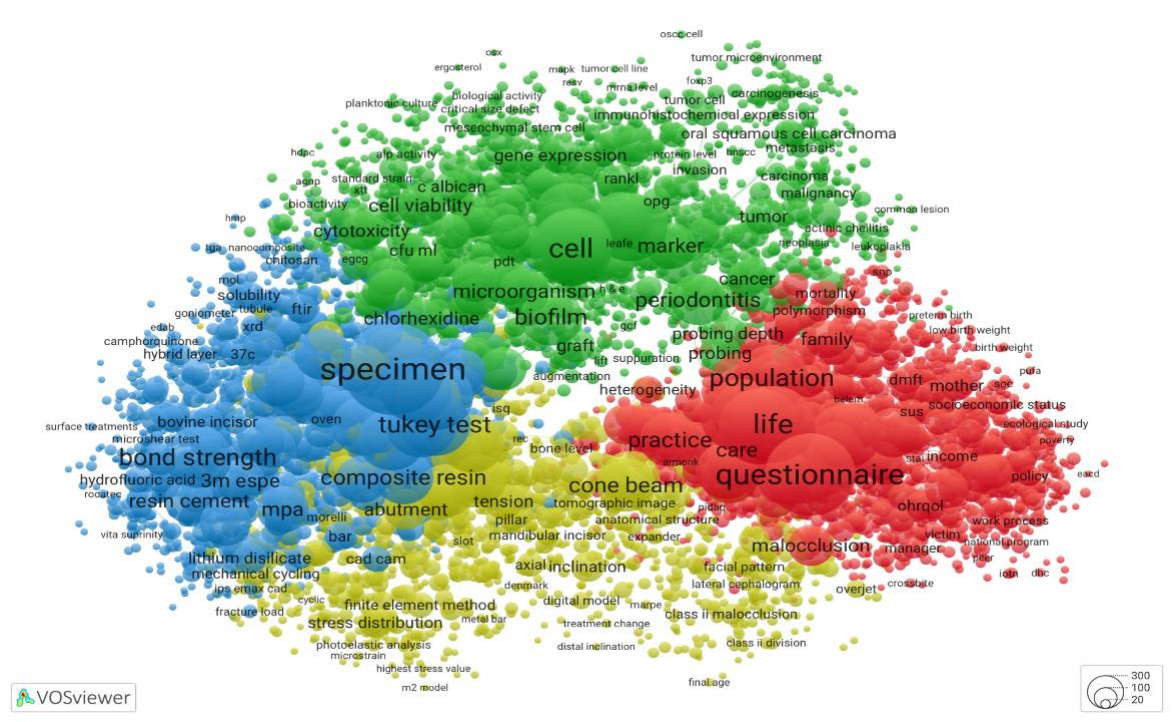
Distribution of clustering themes of master dissertations and doctoral theses from graduate programs in dentistry, Brazil, 2013-2022. Interactive visualization available at https://tinyurl.com/23ojnbsx

Another way to examine the research output of Brazilian graduate programs in dentistry is by analysing the doctoral theses and master's dissertations presented at the PPG. These works provide valuable insights into the focus areas, methodological approaches, and potential societal impact of graduate research. To perform a preliminary analysis of those outputs, the VOSviewer software was used to produce a bibliometric map of abstracts from theses and dissertations produced by Brazilian graduate programs in dentistry between 2013 and 2022. The dataset included approximately 18,000 publications, from which we extracted terms (including noun phrases). For the quantitative analysis, we applied binary counting, whereby the presence of a term in an abstract was noted regardless of its frequency. This approach yielded over 300,000 terms, and from these, we selected those appearing in at least 10 documents, producing a dataset of 11,359 terms. We then calculated a relevance score for each term and chose the most relevant 60%-a practice recommended for highlighting domain-specific terminology while excluding predominantly linguistic markers. The final sample consisted of 6,815 terms, which are depicted in Figure 1.

Figure 1 shows the mapped terms grouped into distinct clusters. Each circle represents a particular term, and its size corresponds to the number of publications in which that term occurs. The arrangement of the circles reflects co-occurrence patterns: terms that frequently appear together are placed closer to one another, whereas terms that rarely co-occur are spaced farther apart. Clusters of closely positioned terms indicate thematic or conceptual proximity, pointing to everyday research contexts or areas. Conversely, clusters situated more distantly suggest unique or less interrelated topics. This visualisation provides an intuitive overview of the research landscape, thus revealing key trends and interconnections between different fields of inquiry.

Four clusters are identified in Figure 1. The green cluster encompasses topics related to oral biology, encompassing cellular and molecular biology, biochemistry, and oral pathology. The second cluster, shown in blue, captures research on dental materials. The yellow cluster comprises dissertations and theses dealing with oral rehabilitation and orthodontics, including dental implants and novel dental prosthesis technologies. Finally, the red cluster highlights studies performed from a social or population-based perspective. Thus, in these roughly 18,000 Brazilian dissertations and theses, there is clear evidence of diverse potential impacts, with social impact featuring prominently.

In dentistry, scientific knowledge generated through dissertations and theses is highly consistent and diverse in terms of topics. The scientific impact of this knowledge, as measured by bibliometric metrics, positions Brazilian dentistry as a leader compared to other countries. In this regard, assessing the quality of graduate programs may include other relevant dimensions, such as societal impact.

### Exploring methods for assessing the societal impact of research in graduate programs

The societal impact of graduate programs is highly significant, given the substantial public budget investment in the Brazilian Graduate Education System, which has increased both the quantity and quality of human resource training and the generation of scientific knowledge. Consequently, assessing the benefits of this expansion to Society is strategic for informing policymakers, stakeholders, and taxpayers of the real gains of such investments. Although methodological approaches to assessing the societal impacts of research remain relatively scarce and underdeveloped, there has been a growing effort to address this limitation.

A narrative systematic review of models and empirical methods for evaluating the impact of health research programs identified various approaches and frameworks ^5^. At least three were based on the W.K. Kellogg Foundation Logic Model. The most frequently cited model is the Payback Framework ^6^, developed in the 1990s by UK researchers. This model features a logical representation of the research process, along with categories to classify 'paybacks,' i.e., the impacts of health research. Its multidimensional categorisation of paybacks encompasses: Knowledge; Benefits to future research and research use; Benefits from informing policy and product development; Health and health sector benefits; and Broader economic benefits. Within the fourth dimension (Health and health sector benefits), the focus lies on improved health, cost reduction in delivering existing services, qualitative improvements in service delivery, and enhanced equity in service provision ^6^.

Another model, also mentioned in the systematic review ^5^, is the Social Impact Assessment Methods through Productive Interactions (SIAMPI) ^7^. This method is founded on concrete data on the processes that generate social impact-namely, productive interactions and stakeholder engagement. It defines the social impact of scientific research as “the measurable effects of the work of a research group or program or a research funding instrument in a relevant social domain. The effect pertains to human well-being (‘quality of life’) and/or the social relations between individuals or organisations.” Quantitative research and case studies are employed to measure such impacts ^7^. Additionally, some authors propose forming expert panels to conduct qualitative evaluations of the social relevance of scientific research ^5^.

In light of the evident shortage of robust and reliable tools for assessing the social impact of research, the 33-item Community Impacts of Research Oriented Partnerships (CIROP) survey instrument was developed for use by research partnerships that address health or social issues. This model covers a variety of dimensions, including the functions of research partnerships, the types of outputs corresponding to these functions, indicators of the usage of these outputs, midterm impacts, and long-term impacts, such as improvements in overall quality of life. The English version of this instrument demonstrates strong psychometric properties regarding reliability and validity ^8^.

To facilitate comparison between the models mentioned above, Table 1 summarizes their conceptual bases, dimensions assessed, methodological approaches, and typical applications in health research.

However, assessing societal impact is challenging due to the diversity of research unit profiles under evaluation. In Brazil, for example, the primary unit of research evaluation is the graduate program. There are nearly 100 programs in dentistry. Some program prioritizes advancing knowledge through cutting-edge research, while others emphasize different forms of impact. This diversity is both expected and desirable, thus reflecting the spectrum of missions and objectives of each program. The Brazilian system explicitly recognizes these distinctions, structuring graduate education into two modalities: academic programs, which follow more traditional scientific research approaches for training scientists; and professional programs, which are designed to produce more applied outcomes and train personnel for working in other societal sectors such as industry and government. As of the beginning of the new evaluation cycle in 2021, dentistry includes 84 academic programs and 15 professional ones, thus illustrating the sector's commitment to balancing outcomes related to scientific advancement as well as practical benefits for citizens regarding health assistance.

**Figure 2:**
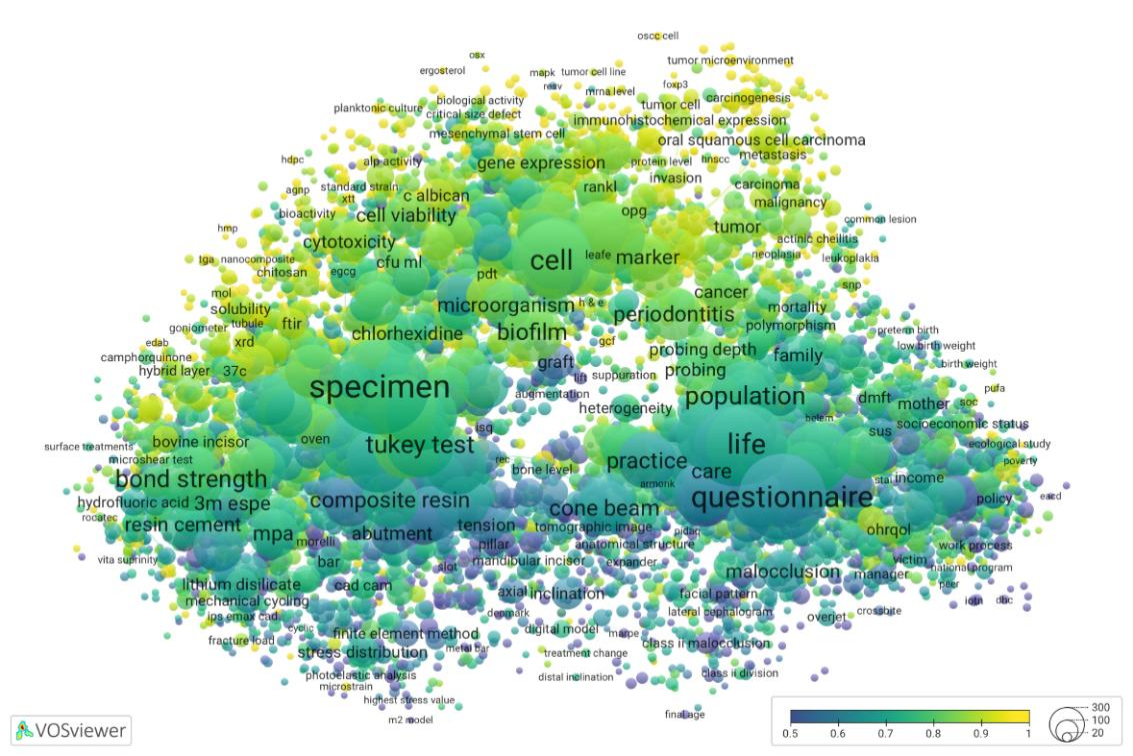
Distribution of clustering themes of academic dissertations and theses from graduate programs in dentistry, Brazil, from 2013 to 2022. Interactive visualization available at https://tinyurl.com/26kkub5g

**Figure 3:**
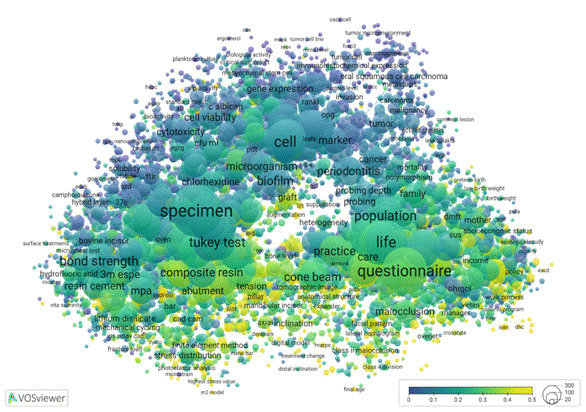
Distribution of clustering themes of professional theses from graduate programs in dentistry, Brazil, from 2013 to 2022. Interactive visualization available at https://tinyurl.com/2ynolqwd

To illustrate the different research profiles of academic and professional programs, we return to the VOSviewer thematic map presented in Figure 1. In preparation for that visualisation from data available in the Open Data CAPES platform, we were able to link each individual graduate program to each abstract in the database, and consequently to the mapped terms in the visualisation. This approach enabled us to label and categorise the data by the originating PPG and thus differentiate between academic and professional graduate programs. By applying a colour overlay to the semantic map, we could visualise the distinct research focuses of these two modalities. The resulting visualisation shows a clear separation in research profiles, with Figure 2 highlighting research in academic programs and Figure 3 showing that of professional ones.

**Table 1:** Comparative summary of frameworks for assessing the societal impact of research

Model	Origin / Basis	Dimensions	Approach	Use Case
Payback	UK, 1990s; linear logic model	Knowledge; Future research; Policy/product influence; Health services; Economic benefits	Mixed (qual/quant)	Widely used in health research impact evaluations
SIAMPI	Netherlands, 2011; “productive interactions”	Focus on interactions between researchers and stakeholders	Qualitative plus case-based	European research funding agencies, including the human sciences
CIROP	Canada; community-based participatory research	Outputs, intermediate, and long-term impacts on community well-being and service improvement	Survey-based (quant)	Community health partnerships and applied social research

Figures 2 and 3 retain the thematic profile presented in Figure 1, differing only in the colour overlays that show the percentage of term occurrences across different program groups. Blueish hues mean lower percentages, light yellow indicates higher percentages, and greenish tones fall in between. In Figure 2, the greatest concentration of research activity appears in the upper clusters, dominated by academic programs, gradually tapering off toward the bottom. Conversely, Figure 3 displays a mirrored pattern, with intensive research focus at the bottom and minimal activity at the top. 

A key distinction lies in the scale ranges of Figures 1 and 2. Academic programs exhibit activity across the thematic spectrum, with their least-involved areas at the bottom still accounting for roughly 50% of occurrences. Oral biology, dental materials, and population research are the most common themes in academic programs. In contrast, the scale for professional programs extends from 0% to 50%, thus indicating that academic programs in fact, also address the seemingly more applied topics. Dental materials and technologies are frequently identified in professional programs. This distribution is expected since professional programs naturally focus on applied research; academic programs are not only focused on advancing basic science concepts, but also contribute to practical applications. As a result, assessing societal impact remains quite relevant for both types of programs, and it is a good point for measuring the interaction of the research environment with Society.

These visualizations, shown in Figures 1 and 2, not only reveal thematic trends but also offer a powerful tool to distinguish the research profiles of academic and professional programs. This comparative lens is especially valuable in the context of Brazil's dual-mode graduate education system, helping to recognize the different missions and contributions of each modality.

**Figure 4:**
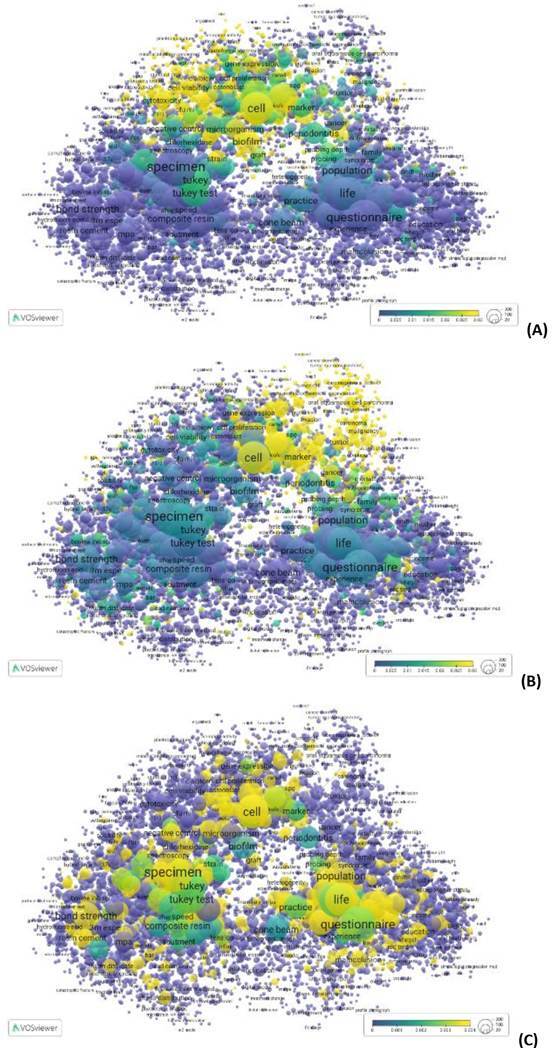
Distribution of clustering themes of theses from three individual graduate programs in dentistry: (a) Program A, (b) Program B, and (c) Program C (2013-2022).

By observing these academic contributions, it becomes possible to understand better how different graduate programs align with scientific advancements or applied outcomes, further illustrating the diversity within the Brazilian graduate education system. This variety is visible even when we look at specific graduate programs, as shown in Figure 4, where we see the thematic focus of three academic programs: (a) Program A, (b) Program B, and (c) Program C.

The analysis of the research profiles of the three graduate programs shown in Figure 4 highlights the variety of research topics across different programs. Figures 4a and 4b illustrate a more concentrated thematic orientation, while Figure 4c presents a broader profile with greater activity in a cluster related to applied research topics. This diversity demonstrates that different research approaches coexist within the graduate education system, each playing a valuable role. A comprehensive impact evaluation should recognise and support this diversity, ensuring that different research profiles are acknowledged and fostered in a way that aligns with their respective missions and contributions to scientific advancement and societal needs.

In summary, the scientific output of Brazilian graduate programs in dentistry reflects high productivity and a growing influence in the global academic community. When interpreted through advanced bibliometric indicators, this output highlights the relevance of these programs in shaping research agendas and informing policy and practice. Combining multiple metrics-both quantitative and qualitative-is crucial to ensure a more nuanced understanding of performance. A robust understanding of scientific impact is, therefore, essential to the development of fair and meaningful assessment models that recognize both scientific excellence and societal contribution.

### A proposal for evaluating societal impact

According to the Kellogg Foundation’s logical model of impact assessment, resources (inputs) drive work processes (activities), which in turn generate products (outputs). These outputs result in outcomes (such as new knowledge, behaviours, and skills) and may lead to both intended and unintended changes in institutions or communities within 7 to 10 years. These changes constitute ‘impacts’ ^9^, which, in the context of this article, refer specifically to the social impacts of graduate programs.

However, as we have seen, investigating different thematic or application-based profiles demands a more complex and multidimensional evaluation process. In this context, the Kellogg model stands to be enriched by a complementary approach that enables a broader selection of indicators. For this reason, our proposal draws on the Strategy Evaluation Protocol (SEP) to incorporate a more multifaceted perspective as we discuss next.

The Strategy Evaluation Protocol (SEP) is a framework established by the Association of Universities in the Netherlands (VSNU), the Netherlands Organisation for Scientific Research (NWO), and the Royal Netherlands Academy of Arts and Sciences (KNAW) to assess research at Dutch universities and research institutes over a six-year cycle. This protocol emphasizes evaluating research units based on their self-defined goals and strategies, promoting a flexible and context-sensitive approach to research assessment.

The Strategy Evaluation Protocol (SEP) 2021-2027 assesses research units based on three main dimensions: research quality, societal relevance, and viability. Research quality focuses on the research's originality, impact, and productivity. Societal relevance evaluates the significance of research contributions to social, economic, cultural, and technological domains. Viability examines the research unit's strategic direction, leadership, and sustainability, ensuring its capacity to adapt and thrive.

To effectively implement the SEP, research units are encouraged to select appropriate qualitative and quantitative indicators that align with their specific goals and strategies. Appendix E of the SEP 2021-2027 provides guidance on choosing these indicators, offering a framework of domains and dimensions to allow for a tailored assessment that reflects each research unit's unique context and objectives. Table 2 shows the current SEP framework of indicators.

**Table 2:** Categories of evidence in the Strategy Evaluation Protocol (SEP) for the quality domains of research quality and relevance to Society.

		Quality domains	
		Research quality	Relevance to Society
Assessment dimensions	Demonstrable products	1. Research products for peers	4. Research products for societal target groups
	Demonstrable use of products	2. Use of research products by peers	5. Use of research products by societal target groups
	Demonstrable marks of recognition	3. Marks of recognition from peers	6. Marks of recognition by societal target groups

Depending on the context of each Graduate Programme, it is possible to identify inputs, outputs, outcomes, and impacts by categorising value activities at local, regional, national, and international contexts. To define social impact, the United Nations' seventeen Sustainable Development Goals can be used as a point of reference ^10^. Specific goals-such as "Health and Well-being" and "Industry, Innovation, and Infrastructure"-are intuitively related to research initiatives in Graduate Programs in Dentistry. However, other objectives may also be achieved through advancements in dental knowledge. For example, developing and disseminating nationally produced dental technologies could help achieve "Decent Work and Economic Growth," contribute to the "Eradication of Poverty," and reduce inequalities. Moreover, emerging insights in dentistry could enhance scientific understanding to inform guidelines for healthier food production or consumption, thereby aiding “Responsible Consumption and Production.” Research endeavours promoting sustainable dental practices could, in turn, affect numerous other United Nations goals.

The methodological approach discussed in this manuscript aims to establish frameworks for assessing the social development contributions of Graduate Programs in Dentistry-from local to regional, national, and international scales. Guided by the United Nations’ Sustainable Development Goals ^10^ and other sources ^5^ ^,^ ^6^ ^,^ ^7^, this proposal recognises the methodological complexities involved and the absence of a validated Brazilian Portuguese instrument, thus demanding an address by a panel of experts ^5^ ^,^ ^11^ ^,^ ^12^ ^,^ ^13^.

In this context, indicators of social impact must be chosen with care to align with the definition above and to be feasible for evaluation within a graduate program over seven years. One strategy could be to propose indicators for "work processes" and "outputs," highlighting their potential to engender wider, long-term outcomes and social impact.

Table 3 presents a proposed set of illustrative indicators aligned with the Sustainable Development Goals (SDGs), the Kellogg Foundation’s logic model, and the Strategy Evaluation Protocol (SEP). These indicators aim to support the monitoring of processes, outputs, and long-term outcomes related to the societal relevance of graduate programs in dentistry.

**Table 3:** Illustrative indicators for assessing the societal impact of graduate programs in dentistry *This flexible set of indicators serves as a reference to be adapted by each program according to its context, goals, and alignment with national health and education policies.*

Indicator	Definition	Sustainable Development Goals	Dimension from the Kellogg Foundation	Dimension from SEF
1.Dissertations or theses on health inequalities	Number of dissertations or theses aimed at describing or analyzing health inequities	Good health and well-being	Output	Research products for societal target groups
2. Published manuscripts on health inequalities	Number of scientific articles on the description and factors associated with the health inequalities	Good health and well-being	Output	Research products for societal target groups
3. Public health actions that use oral health inequality indicators developed by PPGs	Public health programs at the municipal, state, or national level that have used indicators of health inequities for managing healthcare	Good health and well-being	Outcome	Use of research products by societal target groups
4. Public health actions that used oral health inequality indicators developed by PPG, with a record of improvements for the population	Public health programs at the municipal, state, or national level that have used health inequality indicators to organize health care, with improvements in health conditions and quality of life	Good health and well-being	Societal impact	Marks of recognition by societal target groups
5. Dissertations or theses on water quality and sanitation	Number of dissertations and theses evaluating water quality, fluoride heterocontrol, and sanitation	Clean water and sanitation	Output	Research products for societal target groups
6. Published manuscripts on water quality and sanitation	Number of published papers evaluating water quality, fluoride heterocontrol, and sanitation	Clean water and sanitation	Output	Research products for societal target groups
7. Usage of criteria for assessing water quality and sanitation by public bodies	Record of the use of the criteria developed by the PPG to assess the quality of water and sanitation, with a record of improvements in the service provided.	Clean water and sanitation	Societal impact	Marks of recognition by societal target groups
8. Outreach activities with clear interaction with Society driven by research outputs of the PPGs.	University outreach actions with clear interaction with Society - results of research produced by the PPG	Good health and well-being	Output	Research products for societal target groups
9. Improvement in normative health indicators	Advances in reliability, validity, and/or simplification of oral disease indicators.	Good health and well-being	Output	Research products for societal target groups
10. Improvement in quality of life indicators and other patient-reported outcomes	Advances in reliability, validity, and/or simplification of quality of life indicators and other patient-reported outcomes.	Good health and well-being	Output	Research products for societal target groups
11. Developing technology to advance public health communication	Description of the technology developed by the PP	Good health and well-being	Output	Research products for societal target groups
12. Enhanced community health literacy	Record of increased health literacy	Good health and well-being; Quality education	Societal Impact	Marks of recognition by societal target groups
13.Technologies to reduce the cost of dental care in the Brazilian National Health System (SUS)	Description of the technology developed by the PPG to reduce the cost	Decent work and economic growth	Output	Research products for societal target groups
14. Reducing the cost of dental care in the SUS	Record of cost reduction	Decent work and economic growth; Industry, innovation, and infrastructure	Societal Impact	Marks of recognition by societal target groups
15. Technologies developed in the PPG to expand access to oral health services	Description of the technology developed by the PPG to expand access.	Decent work and economic growth; Industry, innovation, and infrastructure	Output	Research products for societal target groups
16. Increased access to oral health services through the use of technology generated in the PPG	Record of increased access to oral health services	Decent work and economic growth; Industry, innovation, and infrastructure	Societal Impact	Marks of recognition by societal target groups
17. Results of dissertations/theses that generate instructional resources	Number of dissertations and theses on this topic	Quality education	Output	Research products for societal target groups
18. Training with instructional resources resulting from research produced by the PPG	Number of trained dentists and oral health teams	Quality education	Output	Research products for societal target groups
19. Work process transformations with indications of improvement stemming from previous training	Record of work process transformations	Good quality work and economic growth	Societal Impact	Marks of recognition by societal target groups

**Supplementary Figure 1:**
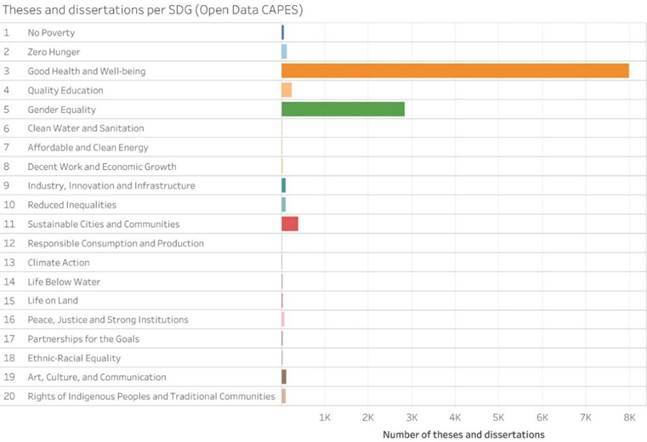
Distribution of theses and dissertations from Brazilian graduate programs in dentistry, according to the SDG (Open Data CAPES, 2013-2022).

**Supplementary Figure 2:**
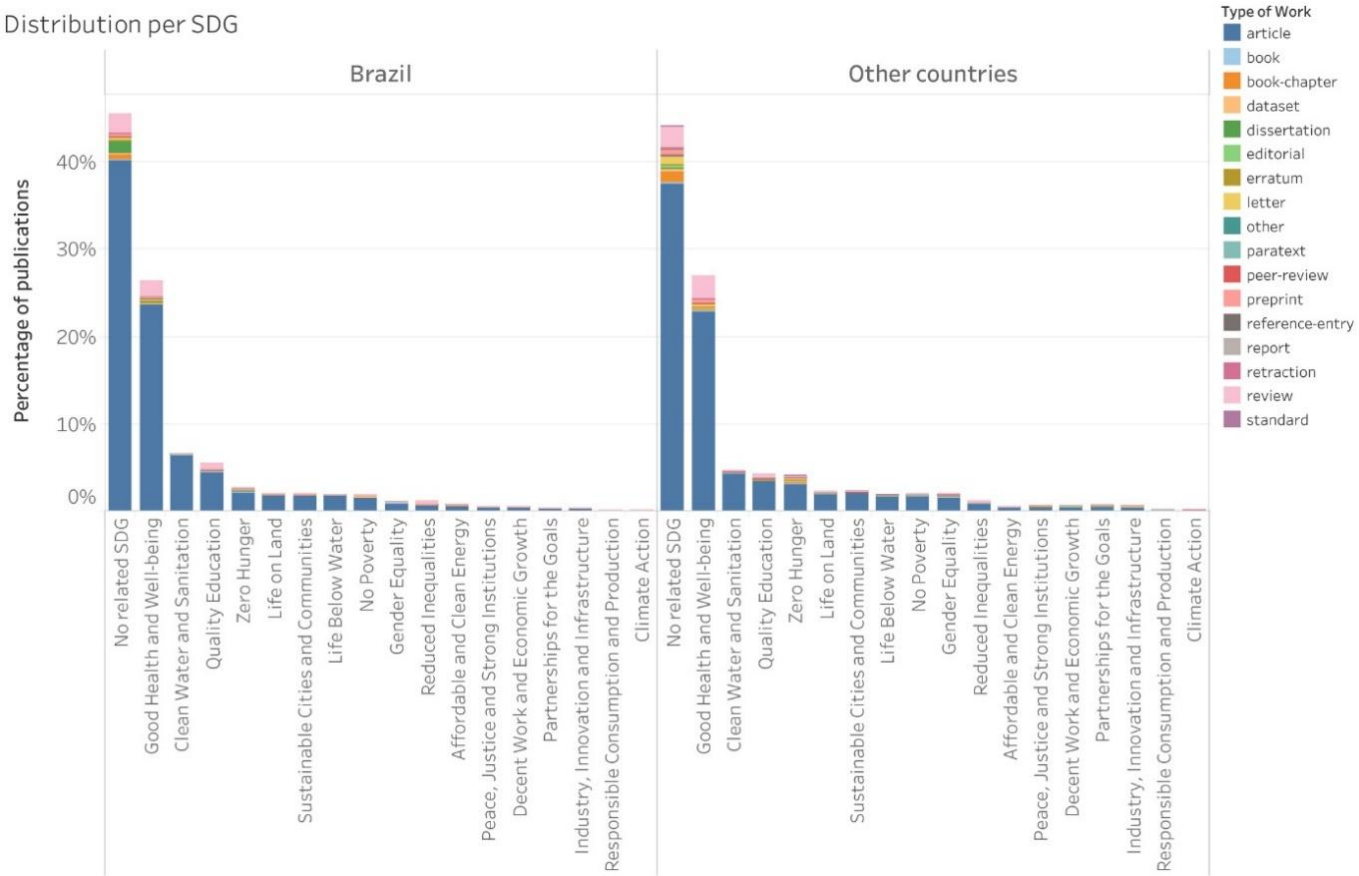
Distribution of dental research publications from Brazil and other countries, according to their relation to the SDG (OpenAlex, 2013-2022).

To further illustrate the alignment between dental research and the SDGs, additional visualizations based on OpenAlex (scientific articles) and the CAPES open database (theses and dissertations) are presented in Supplementary Figures 1 and 2.

## Concluding remarks

The growing demand for more meaningful evaluation frameworks in graduate education requires a multidimensional approach capable of capturing both scientific excellence and societal relevance. In dentistry, the evaluation of graduate programs has historically focused on scientific output, a tradition that has positioned Brazil as a global leader in the field. However, incorporating societal impact remains a grand challenge in the improvement of assessment models.

The proposed set of indicators-aligned with the Sustainable Development Goals (SDGs), the Kellogg Foundation logic model, and the Strategy Evaluation Protocol (SEP)-offers a flexible starting point for monitoring social impact at different levels. It is important to acknowledge that there is currently no validated and standardized model for societal impact assessment developed by either Brazilian research groups or by evaluation and funding agencies. Thus, further refinement and validation-through both psychometric testing and qualitative analysis-are essential to ensure applicability across different program profiles.

In parallel, graduate programs are increasingly required to justify their scientific products qualitatively. Extending this practice to include case studies on socially impactful products could enrich understanding of how research initiatives engage with societal actors and generate real-world benefits. The literature emphasises the need for methodological rigor and contextual grounding in such approaches ^14^.

Articulating impact evaluation with public health policies-such as the National Strategic Action Plan for Non-Communicable Diseases (DANTs 2021-2030) and the strategic priorities of the Brazilian Ministry of Health-can reinforce the alignment between academic output and national development goals. The diversity of themes found in theses and dissertations, as well as the coexistence of academic and professional modalities, underscores the importance of adaptable frameworks that respect each program's mission.

Summing up, Brazilian graduate programs in dentistry have both the scientific capacity and the institutional mandate to drive a positive change in population health. Making this impact visible, measurable, and valued by evaluation systems is essential to support their full transformative potential.

## Footnotes

1 As there are variations in nomenclature across countries, in this paper, we use the term graduate education to refer to master’s programs (PhD) courses. Additionally, we use the term graduate programs (referred to by the Portuguese acronym PPG) to describe teaching and research units offering master’s and/or doctoral courses.

2 Available at: https://www.gov.br/pt-br/noticias/educacao-e-pesquisa/2021/02/pos-graduacao-brasileira-cresceu-48-na-ultima-decada
